# Early life vaccination: Generation of adult-quality memory CD8+ T cells in infant mice using non-replicating adenoviral vectors

**DOI:** 10.1038/srep38666

**Published:** 2016-12-08

**Authors:** Loulieta Nazerai, Maria R. Bassi, Ida E. M. Uddback, Peter J. Holst, Jan P. Christensen, Allan R. Thomsen

**Affiliations:** 1 Department of Immunology and Microbiology, University of Copenhagen, Copenhagen, Denmark

## Abstract

Intracellular pathogens represent a serious threat during early life. Importantly, even though the immune system of newborns may be characterized as developmentally immature, with a propensity to develop Th2 immunity, significant CD8+ T-cell responses may still be elicited in the context of optimal priming. Replication deficient adenoviral vectors have been demonstrated to induce potent CD8+ T-cell response in mice, primates and humans. The aim of the present study was therefore to assess whether replication-deficient adenovectors could overcome the risk of overwhelming antigen stimulation during the first period of life and provide a pertinent alternative in infant vaccinology. To address this, infant mice were vaccinated with three different adenoviral vectors and the CD8+ T-cell response after early life vaccination was explored. We assessed the frequency, polyfunctionality and *in vivo* cytotoxicity of the elicited memory CD8+ T cells, as well as the potential of these cells to respond to secondary infections and confer protection. We further tested the impact of maternal immunity against our replication-deficient adenoviral vector during early life vaccination. Overall, our results indicate that memory CD8+ T cells induced by adenoviral vectors in infant mice are of good quality and match those elicited in the adult host.

The immune response to viral infection represents the result of a complex interaction between the virus, its target cells and several cell subsets belonging to the immune system. There exist a number of differences in the innate and adaptive immune system between infants and adults, and these differences are obvious with regard to the responses elicited by vaccination and infection^1^,^2^.

Viral clearance and disease prevention typically require a combination of humoral and cell mediated immunity. It has been suggested that, while antibodies (Abs) are a correlate of protection against (re) infection, T cell immunity is a correlate of protection against primary disease and persistent infection^3^,^4^. For effective viral clearance, the induction of CD8+ cytotoxic T lymphocytes is often essential and in early life CD8+ T cell responses have been suggested to be impaired and delayed^5^.

T-cell responses elicited in early life have been found to differ from those induced in adult life in terms of numbers, diversity of T cell repertoire, and responsiveness to TCR stimulation^6^. Functionally, there is an impaired induction of cytotoxic T cells and an increased Th2 differentiation leading to increased production of IL-5 and an increased IgG1/IgG2a ratio, while the capacity to produce IFN-γ is reduced^7^,^8^,^9^. Until 1996, the neonatal period was considered a period in ontogeny during which the immune system was immature and prone to tolerization. However, in that year, three studies demonstrated that what was previously believed to represent T-cell tolerance, in fact reflected Th2 type immunity. It was further revealed that inoculation of low doses of murine retrovirus led to the induction of a protective CTL response, and that absence of a CTL response in high-dose infected mice was not the result of immunological immaturity, but correlated with the induction of a non-protective type 2 cytokine response^10^,^11^,^12^. Nevertheless, even today, the mechanism(s) underlying the difference in immune response profile of infants and adults are not absolutely clear. However, delayed maturation of certain DC types leading to limited IL-12 and type I IFN production combined with the fact that the Th2 cytokine locus is epigenetically poised for production of IL-4 and IL-13 may be part of the explanation for the Th2 bias in neonatal immunity^13^,^14^.

The presence of maternal antibodies (Abs) during the first period of life has also been found to represent a critical factor that further complicates early life vaccination^15^,^16^. Circulating Abs, e.g. in the form of maternal Abs, may in theory both augment and inhibit Ab-responses. When Abs are present, non-living antigen may form immune complexes and activate complement, and this may differentially impact antigen uptake and presentation in various types of APCs^16^. Immune complexes may directly inhibit B-cell activation through FcγRIIB-mediated inhibitory signals^17^. On the other hand, complement split products (C3d) may act as an adjuvant and improve immune responses^18^,^19^,^20^. Regarding CD8+ T cell responses, these typically require live vectors, and circulating Abs may reduce CD8+ T-cell mediated immunity by inhibiting vector replication^21^. For that reason human vaccination with current live-replicating attenuated vaccines (i.e. MMR vaccine) is postponed until serum levels of maternal Abs have declined to very low values. Nevertheless, it has been demonstrated that even when maternal Abs have decreased to non-detectable levels, they might still inhibit vaccination attempts with replicating viruses, leaving the infant vulnerable and unprotected against infectious diseases^22^.

Adenoviral vectors are notorious for their inherent capacity to induce strong and long-lasting CD8+ T cell responses against a delivered antigen. The vaccination potential of these vectors may be further improved by linking the encoded antigen to li (MHC class II-associated invariant chain), resulting in an accelerated, augmented and prolonged CD8+ T cell response^23^. Studies so far have established vital knowledge regarding the use of adenoviral vectors for vaccination purposes in mice, non-human primates and humans. However, these studies have mainly been conducted in immunologically mature, adult hosts. In the present study, we have taken advantage of the capacity of adenovectors to elicit CD8+ T-cell responses and employed them to try to induce efficient CD8+ T-cell mediated immunity in mice 10–14 days old, hence forth called infant mice. A critical issue in this context is finding the optimal vaccination dose. Even in adult mice, high dose immunization and/or systemic vector dissemination may lead to generation of partly dysfunctional CD8+ T cells^24^,^25^,^26^,^27^. Given that infant mice are smaller and have fewer T cells, one might predict that lower doses should be used to immunize these mice compared to adults. Quite surprisingly this was not the case.

As our primary model we chose to work with the lymphocytic choriomeningitis virus (LCMV) infection system, exploring the CD8+ T cell response after early life vaccination with Ad-liGP; an adenovector-based vaccine encoding the glycoprotein (GP) of LCMV tethered to Ii. It is of interest that the choice of this model allows us to directly compare the generation of CD8+ T cells using a non-replicating adenovector to that induced by “natural” infection of the same age group with live virus. Thus, for many years it has been known that 17–19 days old mice cannot raise a normal CD8+ effector T-cell response and handle LCMV infection appropriately^28^, and more recent studies have documented that in 2-weeks old mice, the LCMV infection follows a protracted course, reflecting an impaired CD8+ T-cell response apparently consequential to delayed plasmacytoid dendritic cell (PDC) maturation and a deficient type I IFN response^5^,^14^. In addition to Ad-liGP, two more adenoconstructs encoding CD8+ T-cell epitopes from Yellow Fever virus (YFV): Ad-YF CME and Ad-YF NS3, were investigated in the context of early life vaccination. Notably, the efficiency of all vaccine induced responses were tested using several different challenge models, all known to reflect CD8+ T-cell mediated protection as validated in one or more earlier studies^29^,^30^,^31^,^32^,^33^,^34^,^35^. Overall, our results illustrate that, at least for murine CD8+ T cells, there appear to be no major intrinsic functional deficiency, and that given optimal activating conditions efficient CD8+ T-cell-mediated immunity may be elicited in infants. On the more translational note, our results suggest that replication-deficient adenoviral vectors might represent an efficient and safe tool to induce critical cell-mediated antiviral immunity in early life. Importantly, unlike live vectors, maternal immunity to the vaccine was not found to negatively impact the induced T-cell response.

## Results

### Establishing the optimal Ad-liGP vaccination dose in infant mice

Total numbers of T cells in neonates and infants are lower than in adults^36^, so the first pertinent question was whether mice immunized as infants would generate fewer memory CD8+ T cells compared to mice immunized as adults.

**Figure 1:**
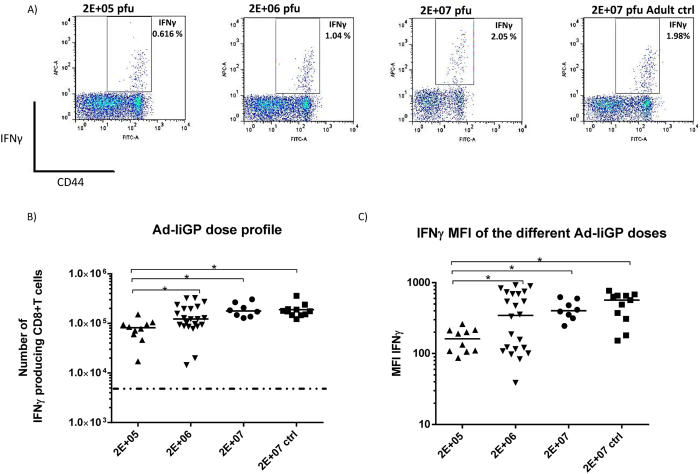
Numbers of GP33 specific memory CD8+ T cells in the spleen as a function of vaccine dose used for early life vaccination. C57BL/6 infant mice were vaccinated with 2 × 10^5^, 2 × 10^6^ or 2 × 10^7^ pfu Ad-liGP s.c in the right hind foot pad and 60 days later IFN-γ producing CD8+ T cells were measured by flow cytometry following *ex vivo* peptide stimulation with GP_33-41_. (**A**) Representative dot plots of the IFN-γ producing CD8+ T cells for each dose. (**B)** Absolute numbers of IFN-γ producing CD8+ T cells in the spleen following vaccination. (**C**) Capacity of stimulated cells to produce IFN-γ as indicated by mean fluorescence intensity (MFI) of IFN-γ producing cells. Mice, vaccinated as adults with 2 × 10^7^ pfu Ad-liGP, were used as positive controls. Each dot represents an individual animal, and the stippled line background. The depicted data are pooled from several independent experiments. *p < 0.05.

However, closely linked to the lower T-cell number in infants, an important question to address was finding the vaccine dose required to elicit the most optimal memory CD8+ T-cell response in infant animals. Even in adult mice too high a vaccine dose may cause CD8+ T-cell dysfunction^24^. Thus, to address the importance of Ad-liGP dose on the magnitude of the elicited memory CD8+ T cell response after early life vaccination, infant mice were vaccinated s.c in the foot pad with different Ad-liGP doses, ranging from 2 × 10^5^ to 2 × 10^7^ pfu, with the latter dose representing the optimal dose in adult mice^37^,^38^. Mice were subsequently evaluated for the number and quality of GP specific CD8+ T cells in the spleen 60 d.p.v. Adult mice, vaccinated with 2 × 10^7^ pfu, were included for comparison to the optimal IFN-γ response. The results (Fig. 1, for specificity controls see Fig. S1), obtained using ICS and flow cytometric analysis, revealed that a dose of 2 × 10^5^ pfu induced a significantly lower CD8+ T cell response than seen in optimally immunized adult mice, whereas vaccination of infants with either 2 × 10^6^ or 2 × 10^7^ pfu resulted in about the same number of splenic memory CD8+ T cells as found in adult controls (Fig. 1B).

These findings excluded the use of the 2 × 10^5^ pfu dose for vaccination of infant mice, due to the very low numbers of GP specific memory CD8+ T cells elicited, and left the other two doses as potential options. To ensure the selection of the dose that would induce the most optimal functional responses in the infants, we next assessed the quality of the generated CD8+ T cells elicited by each dose by comparing the mean fluorescence intensity (MFI) of IFN-γ^+^ cells (Fig. 1C, for representative plots, see Fig. S2). MFI is a good indicator of the amount of IFN-γ released by each antigen-specific CD8+ T cell upon stimulation^39^. Interestingly, while the MFIs of IFN-γ^+^ cells induced by either 2 × 10^6^ or 2 × 10^7^ pfu s.c. did not differ significantly from each other, and both on average matched the MFIs of cells from vaccinated adults, mice vaccinated using the lower dose displayed a less uniform distribution profile, making us hesitant in selecting this dose as the optimal dose for early life vaccination.

Next, we asked whether gender might exert any influence in the elicited immune response, and whether the spread of the response triggered by the 2 × 10^6^ pfu dose could be gender-related (Fig. S3). However, the statistical analysis did not reveal any significant differences, even though female mice vaccinated with 2 × 10^6^ pfu in early life displayed a tendency toward a lower response in comparison to male mice of the same group.

Taken together, the above results indicated that a dose of 2 × 10^7^ pfu of Ad-liGP triggers a more consistent and reliable immune response in mice vaccinated as infants. However, we reasoned that the characteristics of the immune response triggered by 2 × 10^6^ pfu in infants were also worth pursuing, so we continued using both doses for some of the following experiments.

### Memory CD8+ T cells elicited by early life vaccination with Ad-liGP display good proliferative capacity

Having established that the frequency of early life-primed CD8+ T cells can reach adult levels 60 d.p.v with either of the 2 × 10^6^ pfu or 2 × 10^7^ pfu doses, we next wanted to investigate these CD8+ T cells for their ability to rapidly respond to a secondary challenge and proliferate, since efficient clonal expansion is a key functionality of good memory cells.

**Figure 2:**
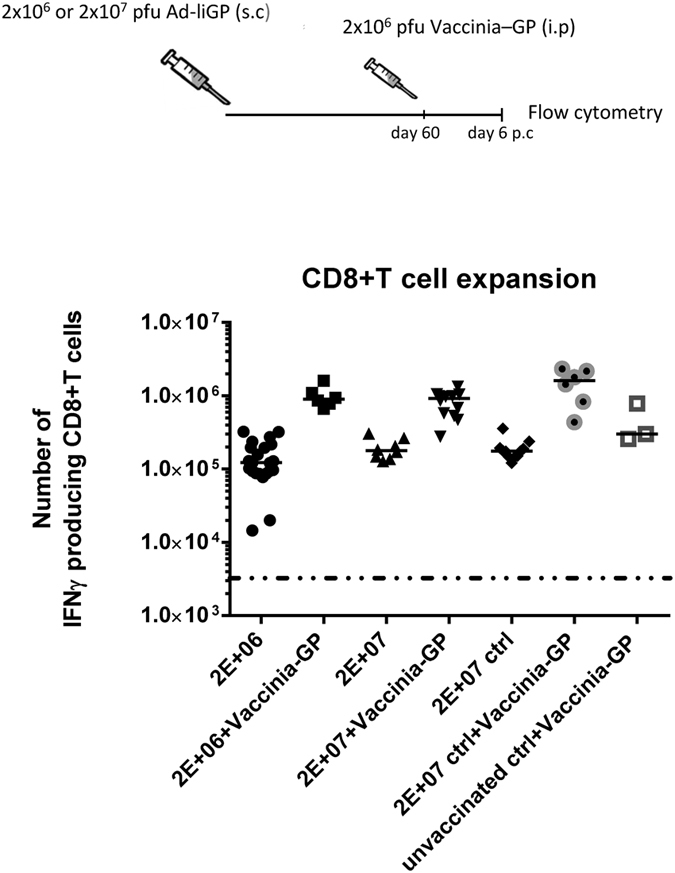
The proliferative capacity of early life induced GP33 specific CD8+ T cells matches that of adult primed cells. C57BL/6 infant mice were vaccinated with 2 × 10^6^ or 2 × 10^7^ pfu Ad-liGP s.c in the foot pad and 60 days later the mice were challenged i.p with 2 × 10^6^ pfu VV-GP. Mice, vaccinated with 2 × 10^7^ pfu Ad-liGP as adults (Ctrl) and similarly challenged, were used as positive controls, and unvaccinated, vaccinia challenged mice were used to illustrate the unprimed/primary VV-GP induced response. Six days post challenge IFN-γ producing CD8+ T cells were enumerated by flow cytometry following *ex vivo* peptide stimulation with GP_33-41_. CD8+ T-cell numbers with and without VV-GP challenge are depicted; the results regarding unchallenged mice are the same as shown in Fig. 1. Each dot represents an individual animal, and the stippled line background. The depicted data are pooled from two independent experiments. *p < 0.05.

To this end, infant mice were vaccinated s.c in the foot pad with 2 × 10^6^ or 2 × 10^7^ pfu Ad-liGP, and 60 d.p.v the mice were challenged i.p with 2 × 10^6^ GP-expressing vaccinia virus (VV-GP)^38^. Six days post challenge, numbers of IFN-γ producing CD8+ T cells in spleen were assessed by ICS and compared to the numbers of IFN-γ producing CD8+ T cells present in vaccinated unchallenged mice (Fig. 2). Mice vaccinated as adults, both challenged and unchallenged, as well as challenged unvaccinated mice, were included for comparison.

We observed that memory CD8+ T cells induced in mice vaccinated as infants and independent of dose (2 × 10^6^ or 2 × 10^7^ pfu Ad-liGP), were able to recognise the GP delivered by the vaccinia vector and were able to expand as efficiently as memory CD8+ T cells from mice vaccinated as adults.

The findings so far indicate that when infants are vaccinated with an appropriate dose of Ad-liGP, a population of long-lived memory CD8+ T cells is generated, which in terms of numbers and proliferative capacity match the memory CD8+ T cells produced following Ad-liGP vaccination in adulthood. This seems to be the case for both 2 × 10^6^ and 2 × 10^7^ pfu Ad-liGP early life vaccination.

### Mice vaccinated with Ad-liGP as infants are protected against acute and chronic viral infection

Most important for an efficient vaccine is the capacity to induce clinical protection. Therefore, we next tested the ability of the Ad-liGP vaccine to induce protection in mice vaccinated as infants by examining the efficiency of the generated CD8+ T cells to control a viral challenge. The fact that both 2 × 10^6^ and 2 × 10^7^ pfu could elicit memory CD8+ T cells that were able to expand in a recall response, made us employ both doses for this further investigation.

For this analysis, infant mice were again vaccinated s.c in the foot pad with either 2 × 10^6^ or 2 × 10^7^ pfu Ad-liGP and left for 60 days to reach memory phase. When at memory phase, groups of mice were challenged with two different strains of LCMV known to induce either an acute or a more persistent infection. Both these challenge models are classical models for evaluation of CD8+ T-cell mediated virus control^31^,^32^,^34^. Furthermore, Ad-IiGP vaccination of adult mice has previously been demonstrated to confer CD8+ T-cell mediated protection^30^.

**Figure 3:**
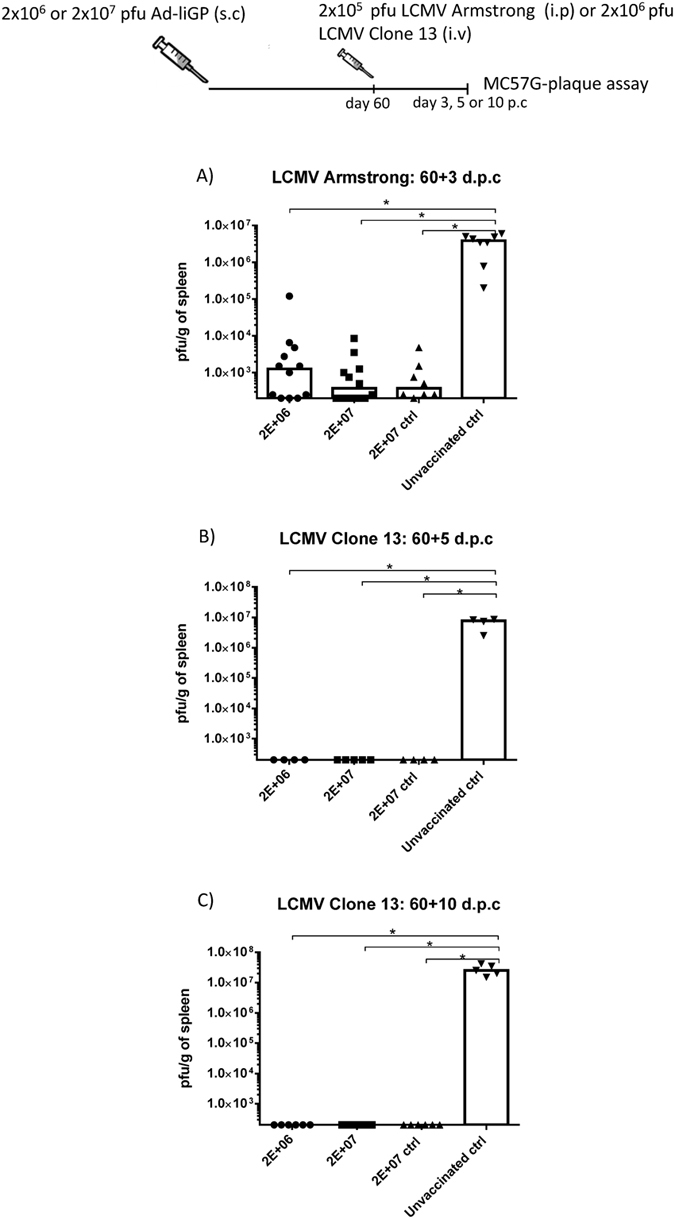
Mice vaccinated in early life can handle both acute and chronic viral infection as efficiently as mice primed as adults. C57BL/6 infant mice were vaccinated with 2 × 10^6^ or 2 × 10^7^ pfu Ad-liGP s.c in the foot pad and 60 days later challenged i.p with 2 × 10^5^ pfu LCMV Armstrong (**A**), 2 × 10^5^ pfu LCMV Clone 13 (**B**) or 2 × 10^6^ pfu LCMV Clone 13 (**C**). Mice, vaccinated as adults with 2 × 10^7^ pfu Ad-liGP (ctrl), and naive unvaccinated mice were similarly challenged and used as positive and negative controls, respectively. Three days **(A)**, 5 **(B)** or 10 (**C**) days post challenge, viral titers in the spleen were measured by plaque assay. Each dot represents an individual animal. The depicted data are pooled from two independent experiments. The detection limit for virus was determined as 250 pfu/g of spleen. *p < 0.05.

To evaluate vaccine induced protection against an acute infection, the Ad-liGP vaccinated mice, as well as a group of naïve mice, were infected i.p with 2 × 10^5^ pfu LCMV Armstrong 53b. Spleens were harvested 3 days later for determination of viral titers. As expected, naïve mice did not control the infection and contained high levels of virus, whereas mice vaccinated as infants with 2 × 10^6^ or 2 × 10^7^ pfu as well as mice vaccinated as adults with 2 × 10^7^ pfu were equally efficient at controlling the infection (Fig. 3A). Interestingly, the splenic viral loads matched the CD8+ T cell response profile for each group (Fig. 1B), with the 2 × 10^6^ pfu-early life-vaccinated mice displaying slightly higher viral loads than the 2 × 10^7^ pfu early life- and adult life-vaccinated mice. Still, the 2 × 10^6^ pfu early life-vaccinated mice were clearly protected compared to naïve mice.

To ascertain that the observed protection reflected CD8+ T-cell mediated immunity, the above experiment was essentially repeated, but in this case half the vaccinated mice, both mice vaccinated as infants and as adults, were depleted of CD8+ T cells prior to challenge. Our results confirmed that CD8+ T cells were the main effector in controlling the infection (Fig. S4A), not only in adults as previously demonstrated, but also in mice vaccinated as infants.

To address the capacity of the vaccinated mice to handle a higher viral challenge dose and a more chronic infection, Ad-liGP vaccinated mice, as well as naïve mice, were infected i.v with 2 × 10^5^ or 2 × 10^6^ pfu LCMV Clone 13 and left for 5 or 10 days, respectively^32^,^34^. Infection of naïve mice with high doses of LCMV clone 13 are known to induce profound CD8 T-cell exhaustion and very high viral loads in the organs around day 10 post infection, thus explaining the choice of time point for analysis regarding the higher dose of clone 13 challenge^34^. Spleens were then collected and the viral loads for each infectious dose were determined. Again, naïve mice were unable to control the infection, whereas the spleens of both early life-vaccinated and adult life-vaccinated mice were found not to contain detectable levels of virus (Fig. 3B,C). Again, we tested whether clinical protection reflected CD8+ T-cell activity by depleting part of the vaccinated mice of CD8+ T cells. Again we observed that CD8+ T-cell depletion eliminated all antiviral activity *in vivo* (Fig. S4B).

Collectively, these data suggest that Ad-liGP vaccination in early life can induce strong anti-viral CD8+ T cell immunity that is translated into remarkable levels of *in vivo* protection against acute and chronic viral infection, similar to that seen in mice vaccinated as adults. Notably, even though both 2 × 10^6^ and 2 × 10^7^ pfu doses were nearly equally efficient in controlling the challenges applied, we decided at this point to work exclusively with the 2 × 10^7^ dose for the rest of the evaluations of Ad-liGP early life vaccination-related parameters.

### Memory CD8+ T cells generated after early life vaccination are functionally similar to memory cells from mice vaccinated as adult

Activated CD8+ T cells employ a number of cellular functions to control viral infections like cytolysis of the infected cells via the granule exocytosis pathway, the initiation of programmed cell death via Fas/FasL interaction and the expression of pro-inflammatory cytokines, such as IFN-γ, TNF-α, IL-2 and certain chemokines. Vaccine-induced protection is traditionally correlated with these effector functions.

**Figure 4:**
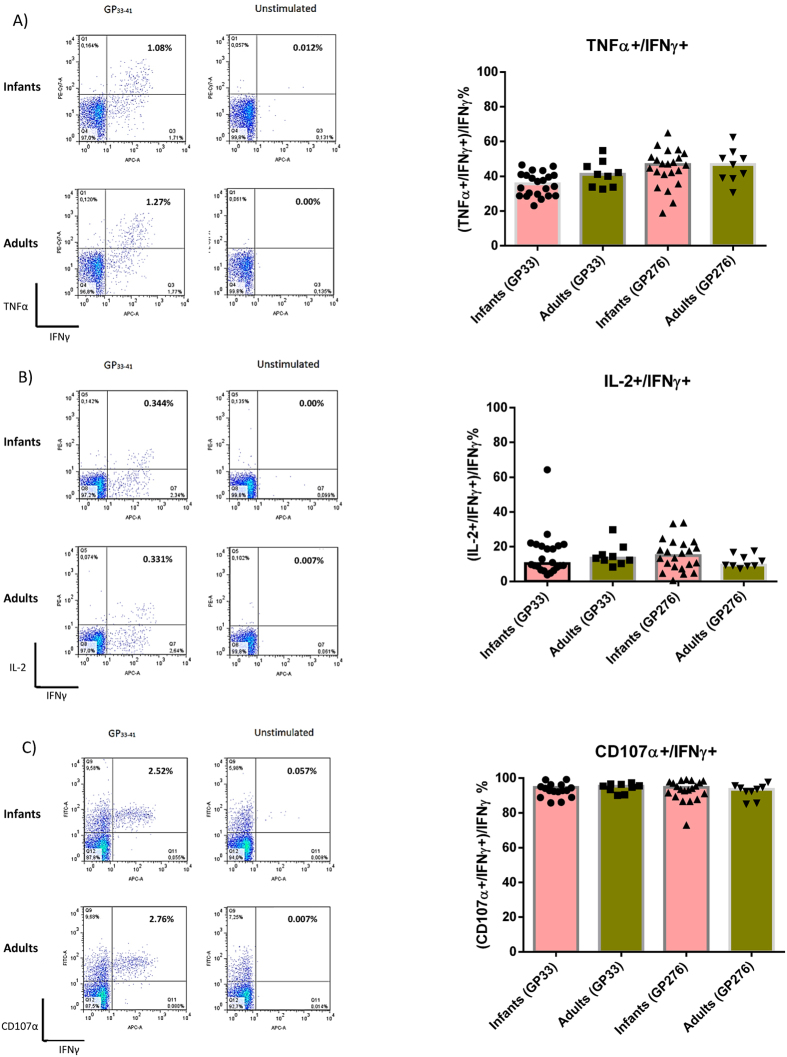
The functional capacity of CD8+ T cells in Ad-liGP vaccinated infant mice is comparable to adults. C57BL/6 infant mice were vaccinated with 2 × 10^7^ pfu Ad-liGP s.c in the foot pad and 60 days later the IFN-γ producing CD8+ T cells co-expressing TNF-α **(A)**, IL-2 **(B)** or CD107α **(C)** were measured by flow cytometry following *ex vivo* peptide stimulation with GP_33-41_ or GP_276-286_. Mice, vaccinated with 2 × 10^7^ pfu Ad-liGP as adults, were used as controls. Left hand side depict representative plots, while the graphs on the right hand side show compiled results from all mice in each group. Each dot represents an individual animal, and bars the averages of the test groups. The depicted data are pooled from two independent experiments. *p < 0.05.

Thus, in order to confirm that the observed *in vivo* protection in Ad-liGP vaccinated mice mirrors polyfunctionality and the ability of the elicited CD8+ T cells to undergo degranulation, infant mice were vaccinated s.c in the foot pad with 2 × 10^7^ pfu Ad-liGP and left for 60 days. Upon reaching the memory phase, splenocytes were harvested and the CD8+ T cells were examined by ICS for their ability to produce TNF-α, IL-2 and express CD107α in addition to IFN-γ production. Degranulation was assessed by incubating the splenocytes with the α-CD107α mAb during the 5h peptide stimulation period of the intracellular staining protocol. We observed that mice vaccinated as infants or as adults were equally capable of producing CD8+ T cells, which were double positive for IFN-γ and TNF-α secretion (TNF-α^+^ IFN-γ^+^) (Fig. 4A), IFN-γ and IL-2 secretion (IL-2^+^ IFN-γ^+^) (Fig. 4B), or IFN-γ secretion and degranulation (CD107α^+^ IFN-γ^+^) (Fig. 4C). Both in mice vaccinated as infants and as adults, antigen-specific cells producing IL-2, also frequently produced TNF-α (Fig. S5), indicating that vaccine-elicited cells included polyfunctional CD8+ T cells. In this set of experiments we extended the analysis to include an additional GP epitope, GP276. This was to ascertain that the observed results were not unique to GP33 specific CD8+ T cells. As can be seen from the data, completely similar results were obtained, irrespective of the epitope analyzed.

In addition to testing *in vitro* functionality of the induced memory CD8+ T cells, we also compared the capacity of these cells to kill antigen expressing target cells in a standard *in vivo* cytotoxicity assay. Specifically, splenocytes from CD45.1 mice were pulsed with either relevant peptide (GP_33–41_) or an irrelevant peptide. Splenocytes loaded with GP_33-41_ were also labeled with CFSE while cells loaded with the irrelevant peptide were left unlabeled. CD45.2 mice vaccinated 60 days earlier, along with a group of naïve CD45.2 mice, were injected i.v with equal numbers of donor cells loaded with relevant peptides and cells loaded with irrelevant peptide. After 16 hours, spleens were collected and flow cytometry was used to quantify the recovery of donor derived cells; specific cytotoxicity was calculated according to the formula described in Materials and Methods. We observed that the *in vivo* cytotoxicity, calculated as the shift in the ratio of relevant peptide-loaded cells to irrelevant peptide-loaded cells, was similar for both early life- and adult life-vaccinated mice (Fig. S6).

### The primary CD8+ T-cell response in Ad-liGP-vaccinated infant mice follows the same kinetics as in adult-vaccinated mice

Thus far the data indicate that early life vaccination with Ad-liGP can be as efficient as adult life vaccination in inducing efficient CD8+ T-cell memory, and seemingly contradicts the notion that a low number of T cells or poor T-cell quality represent an insurmountable restriction to the induction of solid CD8+ T cell mediated immunity in early life. However, how the kinetics of CD8+ T-cell expansion following early life vaccination compares to that in mice vaccinated as adults is not clear from the above studies.

**Figure 5:**
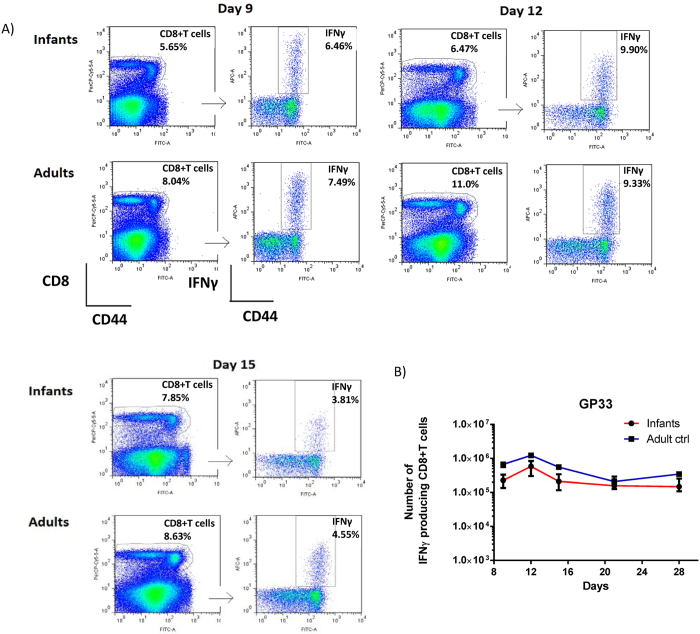
Similar kinetics of the primary GP33 specific CD8+ T-cell response in Ad-liGP-vaccinated mice regardless of age at vaccination. Groups of C57BL/6 infants and adult mice were vaccinated with 2 × 10^7^ pfu Ad-liGP s.c in the foot pad, and on the indicated days following vaccination, spleens were harvested and IFN-Y producing CD8+ T cells in spleen were enumerated by flow cytometry following *ex vivo* peptide stimulation with GP_33-41_. For each time point 5 animals/group of mice vaccinated as infants and 2 animals/group of mice vaccinated as adults were analyzed. (**A**) Representative dot plots for the CD8+ T-cell population and IFN-Y producing cells for days 9, 12, 15 post vaccination. (**B**) absolute numbers of IFN-γ producing CD8+ T cells in the spleen. The results represent the group medians with ranges and are representative of two independent experiments. *p < 0.05.

Consequently, we wanted to monitor the course of the primary CD8+ T cell response after early life vaccination, and compare it to that following adult vaccination. For this purpose, infant and adult mice were vaccinated s.c in the foot pad with 2 × 10^7^ pfu Ad-liGP and the number of antigen specific CD8+ T cells in the spleen was determined at several time points between day 9 and day 28 (Fig. 5A+B). We observed that for both groups the CD8+ T-cell response peaked on day 12 post vaccination, and even though, for each time point tested, numbers of GP specific CD8+ T cells demonstrable in spleen were slightly lower following early life vaccination, we did not observe any major delay in CD8+ T-cell expansion in these mice compared to mice vaccinated as adults. Accordingly, early life vaccination would be expected to induce clinical protection with about the same delay as adult vaccination.

### Ad-liGP vaccinated infant mice are able to handle acute and chronic viral infection as early as 30 days following vaccination

So far, the evaluation of the Ad-liGP -related immune responses has been conducted on day 60 post vaccination. Based on the results in the previous section, we wondered how well mice vaccinated as infants would be able to control a virus challenge already at 30 days post vaccination.

**Figure 6:**
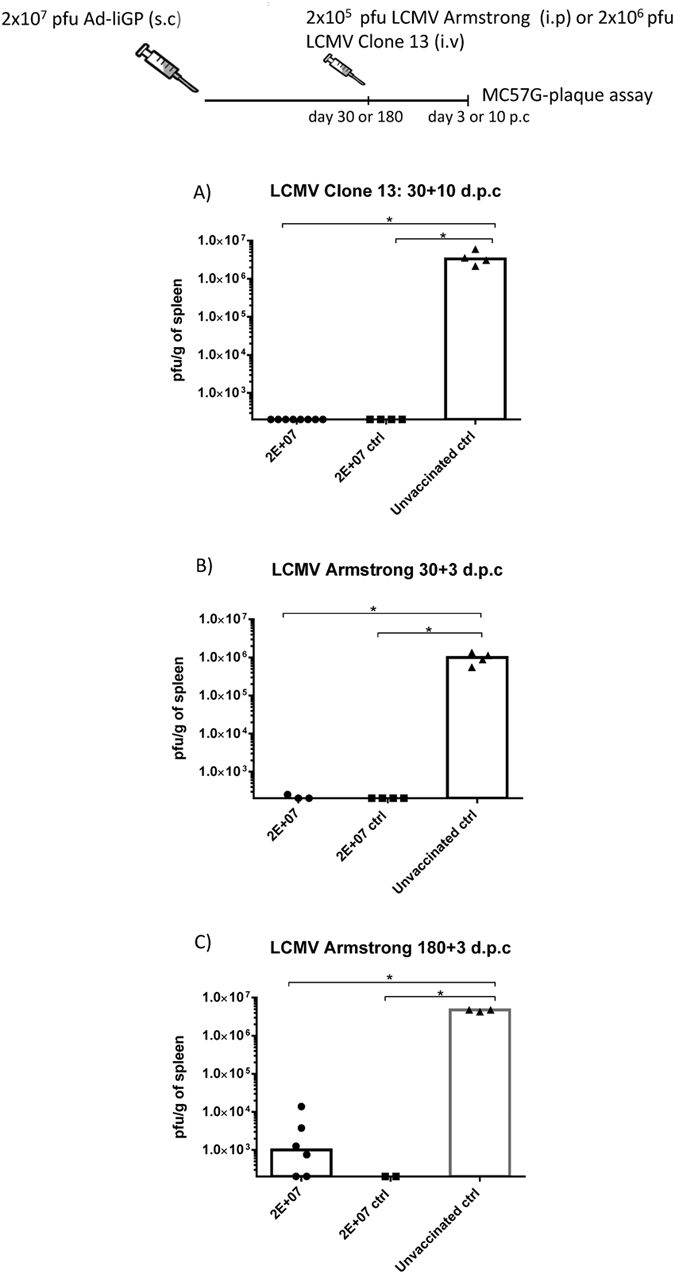
Ad-liGP vaccinated infant mice are protected against LCMV infection both on day 30 and 180 post vaccination. C57BL/6 infant mice were vaccinated with 2 × 10^7^ pfu Ad-liGP s.c in the foot pad. **(A)** 30 days after vaccination mice were challenged i.v with 2 × 10^6^ pfu LCMV Clone 13. Ten days post challenge, the viral titers were measured by plaque assay. **(B,C)** 30 or 180 d.p.v mice were challenged i.p with 2 × 10^5^ pfu LCMV Armstrong. Three days post challenge, the viral titers were measured by plaque assay. Mice, vaccinated as adults with 2 × 10^7^ pfu Ad-liGP, and naive unvaccinated mice were used as positive and negative controls respectively. Each dot represents an individual animal. The detection limit for virus was determined as 250 pfu/g of spleen. *p < 0.05.

To address this, infant mice were vaccinated s.c in the foot pad with 2 × 10^7^ pfu Ad-liGP, and 30 days later they were exposed to an acute or a chronic infection. As previously, the determination of the viral loads in the spleens of vaccinated and naïve mice, following challenge with either 2 × 10^6^ pfu LCMV Clone 13 i.v, or 2 × 10^5^ pfu LCMV Armstrong 53b i.p was used to gauge the antiviral protection against an acute and a chronic infection, respectively (Fig. 6A+B). As expected, naïve mice were incapable of controlling the infection in the spleen. In contrast, all vaccinated mice, whether vaccinated during early life or as adults, could consistently control the viral infections, they were asked to handle.

Thus, our results indicate that GP specific CD8+ T cells, present as early as 30 days subsequent to vaccination of infant mice, are able to control both an acute and a more persistent infection successfully.

### Ad-liGP vaccinated mice vaccinated as infants maintain a functionally efficient memory CD8+ T-cell population even 180 days following vaccination

The last part of evaluating the use of Ad-liGP in early life vaccination was to assess the magnitude of the CD8+ T-cell response and vaccine-induced protection at 180 days after vaccination.

**Figure 7:**
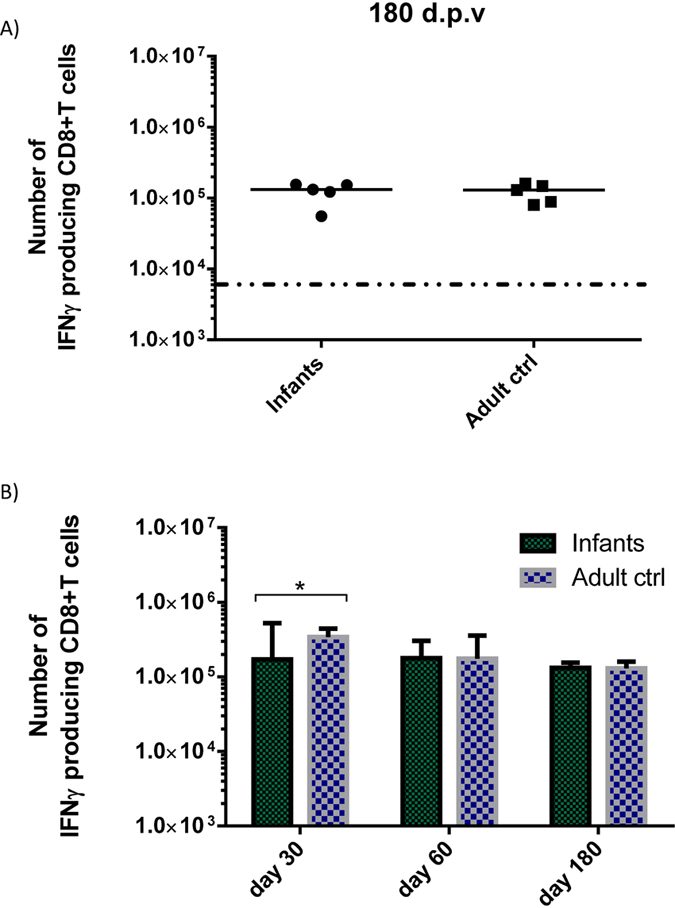
Memory CD8+ T cells in Ad-liGP vaccinated mice are preserved at high levels on day 180 post vaccination regardless of age at vaccination. (**A**) C57BL/6 infants and adult mice were vaccinated with 2 × 10^7^ pfu Ad-liGP s.c in the foot pad and 180 days later numbers of IFN-γ producing CD8+ T cells in the spleen were determined by flow cytometry following *ex vivo* peptide stimulation with GP_33-41_. (**B**) An overview of memory CD8+ T-cell levels as a function of time is also depicted; the results for day 30 and 60 have been taken from previous experiments. Each dot represents an individual animal, and the stippled line background. The results illustrate the group medians with ranges. *p < 0.05.

Therefore, infant mice were vaccinated s.c in the foot pad with 2 × 10^7^ pfu Ad-liGP and 180 days later numbers of IFN-γ secreting CD8+ T cells were assessed by ICS. The results, obtained using flow cytometric analysis, revealed that CD8+ T-cell memory was as stable in early life vaccinated mice as in mice vaccinated as adults (Fig. 7A).

Having established that good CD8+ T-cell memory could be demonstrated in terms of magnitude for as long as 180 d.p.v, we next wanted to gauge this response functionally by testing their ability to protect against viral challenge. Thus, infant mice were vaccinated s.c in the foot pad with 2 × 10^7^ pfu Ad-liGP and 180 days later they were challenged i.p with 2 × 10^5^ pfu LCMV Armstrong. Mice similarly vaccinated as adults and naïve controls were included as controls. Spleens were harvested 3 days later for determination of viral titers (Fig. 6C). In comparison to the high viral titers found in the spleens of the naïve mice, all vaccinated mice were able to contain the infection.

Overall, our results indicate that there is little decrease in the numbers of GP-specific CD8+ memory T cells in the spleens between day 30 and 180 post vaccination (Fig. 7B), suggesting that this CD8+ T-cell memory population is preserved at high levels throughout the time frame studied, for both infant-primed and adult-primed CD8+ T cells. Apart from the stable numbers of memory CD8+ T cells, it also appears that these cells are functionally qualified to confer antiviral protection.

### Mice are protected from lethal bacterial infection following early life vaccination with Ad-liGP

The fact that the Ad-liGP vaccination of adult mice has proved to be highly efficient against viral infections has led this principle to be tested also against non-viral pathogens, such as the intracellular bacteria *Listeria monocytogenes*.

Since we have previously shown that Ad-liGP induced CD8+ T cells protect adult mice against recombinant *L. monocytogenes* expressing the GP from LCMV (Lm-GP)^29^, we wished to take this a step further and investigate how early life-vaccinated mice would handle such a challenge.

With that in mind, infant and adult mice were vaccinated s.c in the foot pad with 2 × 10^7^ pfu Ad-liGP and 60 days later, vaccinated mice, as well as a group of naïve mice, were challenged with a lethal dose of Lm-GP and monitored for mortality until day 3 post challenge (Fig. S7).

As expected, the naïve mice were unprotected, with 90% succumbing to infection, while both infant- and adult-vaccinated mice had a mortality rate of about 40%. These results suggest that, also in this model, early life vaccination with Ad-liGP confers the same levels of protection against a lethal infectious challenge as adult vaccination.

### The CD8+ T-cell responses triggered by vaccination of infant mice with Ad5-YF vectors are comparable to those of mice vaccinated as adults

**Figure 8:**
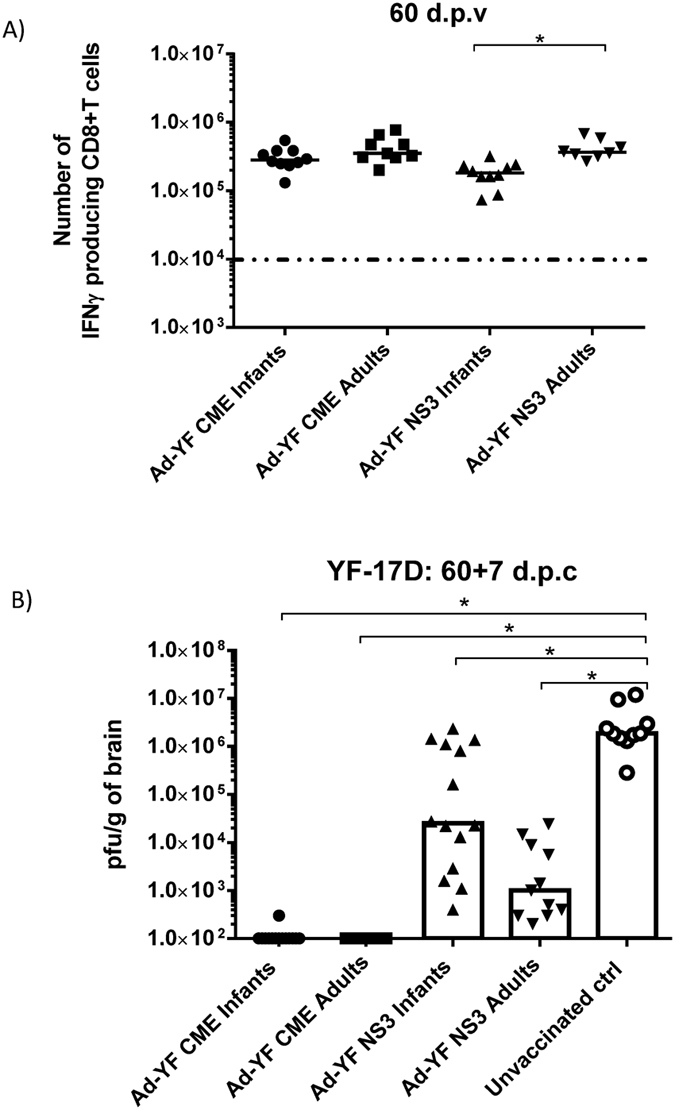
Analysis of immune responses induced by early life vaccination with Ad-YF vectors. C57BL/6 infant mice were vaccinated with 2 × 10^7^ pfu Ad-YF CME or Ad-YF NS3 s.c in the foot pad. **(A)** On day 60 numbers of IFN-γ producing CD8+ T cells in the spleen were determined by flow cytometry following *ex vivo* peptide stimulation with NS3_(268-275)_ and E_(4-12)_. Mice, similarly vaccinated with 2 × 10^7^ pfu Ad-YF CME or Ad-YF NS3 as adults, were used as controls. Each dot represents an individual animal, and the stippled line background. **(B)** Vaccinated mice were challenged i.c with 10^4^ pfu YF-17D virus, and 7 days post challenge their brains were removed for titrating of virus content (IFA); unvaccinated, but challenged, mice were included for comparison. Each dot represents an individual animal. The depicted data are pooled from two independent experiments. The detection limit for virus was determined as 100 pfu/g of brains. *p < 0.05.

To expand on the generality of the above results, we sought to evaluate the immunogenicity, after early life vaccination, of two different Ad-YF constructs encoding structural or internal proteins of the YF-17D virus. Recent studies employing the Ad-YF CME construct, expressing the surface antigens C,M,E, and the Ad-YF NS3 construct, expressing the non-structural protein 3, have demonstrated the induction of relevant CD8+ T-cell immunity in adult B6 mice^33^. The first approach to test these vectors for early life vaccination was to evaluate their ability to induce antigen specific memory CD8+ T cells and to compare numbers and quality of these cells to those generated by adult immunization. Consequently, infant and adult mice were inoculated s.c in the foot pad with 2 × 10^7^ pfu of either of the adeno-constructs and were left to reach memory phase. On day 60, numbers of peptide specific IFN-γ producing CD8+ T cells were determined as parameter of the expansion of CD8+ T cells in response to the yellow fever epitopes encoded by either vector. As can be seen in Fig. 8A, the Ad-YF CME construct induced memory T-cell responses of similar magnitude irrespective of age at vaccination. In contrast, the Ad-YF-NS3 vector elicited a slightly lower CD8+ T-cell response in infant mice compared to adults.

### Infant mice vaccinated with either of the Ad5-YF constructs are significantly protected after YF-17D i.c challenge

Having confirmed the immunogenicity of both YF constructs in infant mice, we next evaluated vaccine induced protection. In order to do so, infant and adult mice were vaccinated s.c with 2 × 10^7^ pfu of the construct in question, and 60 days later all animals, plus naïve controls, were challenged i.c with 10^4^ pfu of YF-17D. 7 days later, the viral loads in the brain were investigated.

The determination of viral titers in the brain (Fig. 8B) confirmed previous findings^33^ showing that the unvaccinated challenged mice had high viral loads in the CNS and were unable to clear the infection. No infectious virus was recovered from either group of Ad-YF CME -primed mice; this very efficient virus control probably reflects the combined effect of both CD8+ T cells and neutralizing antibodies as previously demonstrated in mice vaccinated with Ad-CME as adults^33^. In contrast, and in full agreement with our previous studies, the infection was only partly controlled in the Ad-YF NS3-primed mice, and mice vaccinated as infants were not as consistent in their capacity to control the infection as were mice primed as adults (Fig. 8B), matching the slightly reduced levels of memory CD8+ T cells present in these mice prior to challenge (Fig. 8A).

### Maternal immunity to the vector does not interfere with the success of early life vaccination

Assuming that early life vaccination with adenevectors became standard, all pregnant mothers would be immune to the vector, and we have previously found that vector immunity may significantly reduce the vaccine response^37^. Consequently, we decided to study if maternal immunity might impact on the outcome of early life vaccination with replication deficient adenoviral vectors.

**Figure 9:**
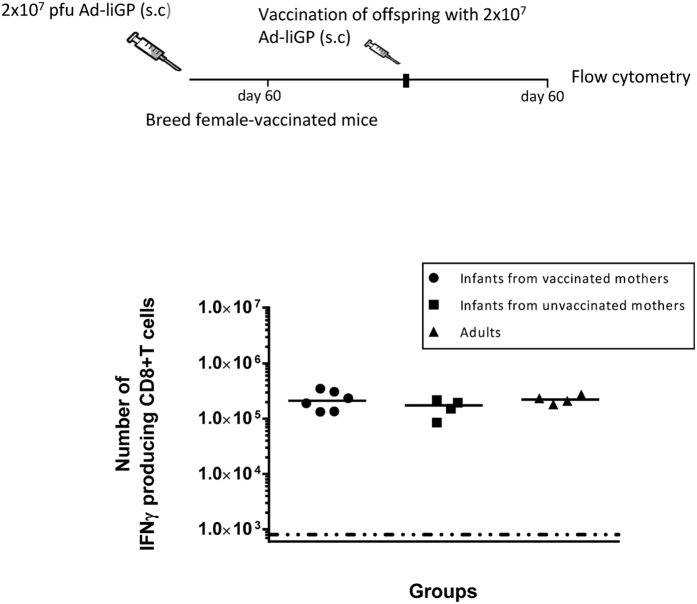
Pre-existing maternal immunity to the vector does not interfere with vaccination efficiency. C57BL/6 infant mice were vaccinated with 2 × 10^7^ pfu Ad-liGP s.c in the foot pad and 60 days later female mice were selected for subsequent breeding. The offspring of these mothers were vaccinated with 2 × 10^7^ pfu Ad-liGP when they reached the age of 10–14 days, and 60 days later GP specific IFN-γ producing CD8+ T cells were measured by flow cytometry following *ex vivo* peptide stimulation with GP_33-41_. Mice, born from un-vaccinated moms and vaccinated with 2 × 10^7^ pfu Ad-liGP as adults, as well as normal mice vaccinated as adults, were used as controls. Results are representative of 2 independent experiments. Each dot represents an individual animal.

For that purpose, 10–14 days old mice were vaccinated s.c. in the foot pad with 2 × 10^7^ pfu Ad-liGP and 60 d.p.v female mice were selected for breeding. When the offspring of the vaccinated-female mice reached the age of 10–14 days, they were themselves vaccinated with 2 × 10^7^ pfu Ad-liGP and 60 d.p.v antigen specific CD8+ T cells in the spleens were enumerated by ICS. The responses induced in these mice were compared to those elicited in mice, born of un-vaccinated moms and vaccinated as infants. Mice vaccinated as adults were also included for comparison. Our results clearly showed that the vaccination status of the mothers did not significantly impact the numbers of IFN-γ producing memory CD8+ T cells generated following vaccination with Ad-IiGP (Fig. 9). These results imply that maternal immunity to the adenovector-based vaccine construct does not pose an obstacle to successful early-life immunization with the same vaccine.

### Significant protection even in mice vaccinated 1 day after birth

**Figure 10:**
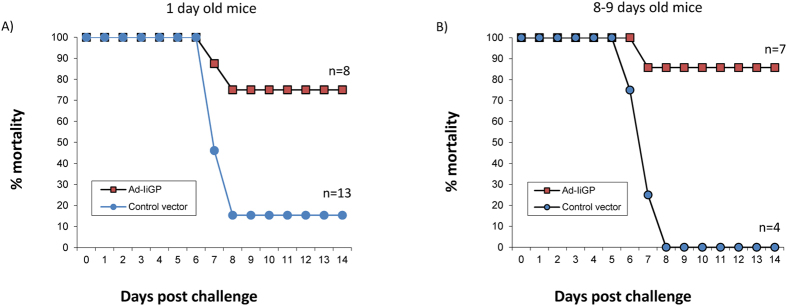
Vaccination with Ad-liGP as early 1 day after birth confers significant protection against i.c. LCMV infection. Newborn (**A**) and infant (**B**) C57BL/6 mice were vaccinated s.c. in the foot pad with 2 × 10^6^ pfu Ad-liGP or a control vector, and 60 days later all mice were challenged i.c with 20 pfu LCMV Armstrong. The mortality of the challenged mice were monitored for 14 days. Results are pooled from several experiments.

Finally, we wanted to see if mice younger than 10–14 days could be significantly protected by adenovector immunization. To this end, mice born 1 or 8–9 days earlier were vaccinated with Ad-IiGP. For these experiments, we chose to reduce the vaccine dose to 2 × 10^6^ pfu of Ad-IiGP, since we already knew that this dose would work well in the “older” mice, and we did not want to overwhelm the immune system in the newborns. Hence, groups of 1 and 8–9 days old mice were vaccinated either with 2 × 10^6^ pfu of Ad-IiGP or a control vector, and 60 days later all mice were challenged i.c. with 20 pfu of LCMV Armstong. Using this route of infection, all naïve mice would die from meningitis, while an accelerated CD8+ T-cell response in vaccinated mice would abort the CNS infection and rescue the mice^24^. As it can be seen in Fig. 10, not only did the majority of mice vaccinated at 8–9 days after birth survive the i.c. challenge - as we expected based on our previous results -, but also mice vaccinated 1 day after birth were significantly protected. Thus, vaccination with Ad-IiGP can be used to induce protection even in 1 day old mice.

## Discussions

Vaccines have been used to prevent disease for more than 200 years and are responsible for many public health successes. However, despite the advances in immunology and molecular biology, viral pathogens like HIV, HBV, EBV and HSV remain a threat to the human population. The infant population is even more vulnerable to viral infections^1^,^2^, and given the distinct immunological features of early life, the design of effective vaccines to be administered in infants becomes even more challenging. The widely held view about early life effector T cells is that they are intrinsically anti-inflammatory, and that their propensity to differentiate into Th2 cells contributes to vaccination failure. Here, we employed a vaccination strategy that is known to induce very potent CD8+ T cell responses in adult individuals to try and tackle the problem of early life infectious diseases. Overall, our findings indicate that replication-deficient adenoviral vectors might represent a promising platform for early life vaccination.

In most of our studies we used the Ad-liGP vector for vaccination of infant mice, and compared the elicited GP specific CD8+ T cell response to that of mice similarly vaccinated as adults. Because infant mice contain fewer T cells than adults^36^, and virus dose is a concern even in adult mice vaccinated using adenovectors^24^,^26^, we started by establishing the vaccine dose required for optimal vaccination of infant mice. Through testing of three different doses, we found that the best and most consistent results were elicited using the same dose also found to be optimal in adult mice, 2 × 10^7^ pfu^37^. Thus, quite surprisingly, there is no apparent need for a dose adjustment related to vaccination age, which is quite practical both when comparing results from mice vaccinated at different ages and when designing vaccine programmes for real-life. Moreover, we observed that the induced responses were not influenced by gender. Taking into account that a number of studies have shown gender-specific effects regarding the impact of vaccination in infancy^40^,^41^,^42^, this makes the use of adenovectors even more appealing.

Interestingly, we noted that, amongst the doses tested, also 2 × 10^6^ pfu induced a significant immune response. However, when we analysed the MFI of the IFN-γ signal in stimulated CD8+ T cells, we observed a substantial interindividual spread, with half of the population displaying an adult quality T-cell response, while the other half displayed substantially lower levels of MFI (Fig. 1C). This might reflect the existence of a threshold of responsiveness, which is not crossed in all infants vaccinated using this dose. Since consistency is clearly an important issue in vaccination, we preferred to use the 2 × 10^7^ dose for most of our experiments.

When tested for their ability to expand following challenge with VV-GP, infant-primed CD8+ T cells were found to have the same proliferative capacity as adult-primed CD8+ T cells. Moreover, apart from good expansion upon a recall challenge, the generated infant-primed memory CD8+ T cells were also functionally apt at conferring protection as tested against both an acute and a more persistent infection. Thus, it seems that early life vaccination with Ad-liGP induced memory CD8+ T cells that are able to respond and handle a viral invasion as efficiently as adult primed cells, both when it comes to speed of virus control and in the context of a more sustained antigenic challenge.

A recent study from Rudd *et al*., suggested that due to the restricted diversity of the T cell repertoire during the first period of life, early life vaccination recruits ‘incomplete’ clonotypes that, even though they transit normally into the adult memory pool, exhibit restricted recall response during a secondary challenge, and confer reduced immunity^43^. The reason why our results are so different from those previously reported, we do not know, but several differences in the set-up should be noticed. First, they used infants that were 7-days old, while we worked with infants between 10–14 days old, this might impact the responsiveness and account for some of the difference. On the other hand, in Fig. 10 we found that mice as young as 1 day old can be significantly protected by adenoviral vaccination. Consequently, the most critical difference is probably that they used live recombinant vectors, and this required a dose adjustment between adults and infants, the consequences of which are hard to predict. Clearly we do not know the TCR repertoire of the cells recruited by our vaccine regimen neither in adults nor in infants, but the question is how important it actually is. Thus, very recently published data indicate that the neonatal TCR repertoire rapidly diversifies during chronic antigen stimulation^44^, as it is believed to be the situation with adenovector vaccination^45^,^46^. Furthermore, there are data, which indicate that differences in TCR composition may be quite effectively compensated by the CD8 co-receptor^47^. Therefore, most important, from a functional point of view we saw little or no difference in the protection afforded by infant and adult primed CD8+ T cells. Consequently, based on the results obtained in our study, compared to recently published data on infection with live LCMV^5^, we conclude that vaccination with replication deficient adenoviral constructs is superior to vaccination with live viruses in early life. The reasons for this are not clear, but it could be speculated that the critical role played by type I IFN both in controlling early viral replication and the differentiation of CD8+ T cells following infection with live virus may play an important role^48^,^49^. Thus, the delayed maturation of PDCs in infant mice seems to critically impair responses to live virus^14^, and even in adult mice absence of IFNAR signalling may completely prevent a CD8+ T-cell response to LCMV (ref. 48 and own unpublished data). In contrast, type I IFN is reported not to be critical to an adenovector induced CD8+ T-cell response (ref. 50 and own unpublished observations have confirmed that this is true also for the response to AdIiGP), and therefore the reduced type I IFN signaling in early life would not impact this response. Alternatively, a difference in the ability of infants and adults in producing IL-33 could play a role; while induction of a CD8+ T cell response to LCMV is critically dependent on IL-33, adenovector immunization is not^51^.

In complete accordance with their *in vivo* performance, memory CD8+ T cells, elicited after early life vaccination with Ad-liGP, matched adult primed CD8+ T cells in their capacity to produce multiple cytokines and degranulate upon antigen recognition *ex vivo*. We could further show that the *in vivo* killing capacity of infant-primed memory CD8+ T cells is comparable to that of memory cells generated in mice vaccinated as adults. Taken together, these findings contradict the widely held notion that early-primed CD8+ T cells are functionally impaired and unable to produce multiple cytokines at the same levels as adults^52^. Indeed, our results support the idea that there are no intrinsic defects in the CD8+ T cells early in life, but rather that their activation is strictly regulated by the stimulating microenvironment^53^, and can be successfully triggered under the appropriate circumstances (see also ref. 54).

We additionally checked the antiviral protection conferred 30 and 180 days after vaccination. We observed that the CD8+ T cells generated as early as 30 d.p.v as well as the CD8+ T cells persisting 180 days after vaccination were both capable of conferring significant antiviral protection (Fig. 6). Furthermore, we compared the magnitude of the elicited CD8+ T cell response at different time-points and found that numbers of virus-specific CD8+ T cells in the spleen on day 180 post vaccination remained as high as on day 60, for both infant-primed and adult-primed CD8+ T cells (Fig. 7). These findings indicate the long term stability of a protective memory population.

In addition to assessing the protection against viral infections, we investigated the protection of early life vaccinated mice against the intracellular bacteria *Listeria monocytogenes*. Jensen *et al*., have previously demonstrated that the vaccination potential of Ad-liGP, in adult hosts, is not confined to viral pathogens but can also be employed against intracellular bacterial infections^29^. Here, we found that Ad-liGP vaccination protects both mice vaccinated as adults and as infants from a lethal bacterial infection.

Besides Ad-liGP, we evaluated the early life vaccination potential of two additional replication deficient Ad5-based vectors encoding YF-17D antigens: one encoding the three viral structural proteins C,M,E (Ad-YF CME) and the other the non-structural protein 3 (Ad-YF NS3). The currently used, live attenuated vaccine against yellow fever, though successfully applied for many years, has displayed a number of vaccine associated adverse effects that have emphasised the need for alternative vaccination strategies. Moreover, the fact that the traditional vaccine cannot be administered to certain groups of the population, including infants, has made it relevant to explore for other vaccination options. Recently, Bassi *et al*., showed that both vectors in question confer antiviral protection when tested in adult mice, and that CD8+ T cells contribute at a great degree to the overall immunity^33^. Both constructs were found to elicit potent memory CD8+ T-cell responses in infants as well as in adult mice, albeit the response to NS3 was slightly reduced in early life primed mice compared to the response in adult mice. Notably, vaccination with the Ad-YF CME vaccine known to induce both neutralizing Abs and CD8+ T cell mediated immunity effectively protected all mice against i.c. infection, while the protection conferred by the Ad-YF NS3 construct that relies exclusively on CD8+ T cells^33^, was not as uniform in mice primed as infants as it was in the case of adult primed mice (Fig. 8B). This interindividual variation correlated well with the slightly reduced numbers of NS3 specific CD8+ T cells in the former mice. The reason for the slight variation in the immunogenicity of different adenoviral vectors in infants versus adults is not known, but clearly it does not merely reflect the presence or not of the immune response enhancer Ii. Perhaps variations in the abundance of relevant peptides for presentation to the T cells in combination with relevant co-stimulatory signals may play a critical role. In this regard it could be of interest to test whether the immunogenicity of NS3 might benefit from li-linkage.

A key obstacle to early vaccination is transferred, pre-existing maternal immunity^16^. For that reason we tested whether maternal immunity would interfere with adenovector immunization in early life, and found no evidence supporting this possibility; this adds one more attractive feature to adenoviral vectors as delivery platform for early life vaccination. In conclusion, our results indicate that there is no major intrinsic functional deficits of infant CD8+ T cells, and that vaccination with non-replicating adenovectors leads to optimal conditions for T-cell priming even in very young individuals. Thus, adenovector immunization could represent a promising and practical solution to the problem of inducing efficient cell mediated immunity in early life.

## Materials and Methods

### Mice

Adult C57BL/6 (CD45.2) female mice, purchased from Taconic Farms (Ry, Denmark) at the age of 6–8 weeks old, were used as controls in all experiments. Infant C57BL/6 (CD45.2) mice were bred locally, from breeding pairs originating from Taconic Farms; these were introduced into experiments when they reached the age of 10–14 days. The breeding cages were checked daily and the precise date of birth was recorded. B6.SJL mice (CD45.1) were bred locally from breeding pairs originating from The Jackson Laboratory (Bar Harbor, ME, USA).

All mice were housed under specific pathogen free (SPF) conditions and the mice from outside sources were allowed to rest for a week before use.

### Ethics statement

Experiments were conducted in accordance with national Danish guidelines (Amendment # 1306 of November 23, 2007) regarding animal experiments as approved by the Danish Animal Experiments Inspectorate, Ministry of Justice, permission numbers 2009/561-1679 and 2015-15-0201-00623.

### Recombinant adenoviral vectors and vaccination

E1-deleted E3 inactivated, human type 5 recombinant adenovirus (Ad5) vectors expressing the GP of LCMV tethered to the MHC class II-associated invariant chain (Ii) (**Ad-liGP**), the yellow fever structural proteins core, membrane and envelope (**Ad-YF CME**), or the yellow fever nonstructural protein 3 (**Ad-YF NS3**) had previously been constructed as described^23^,^33^,^55^. The vaccine solutions were prepared by diluting the adenoviral vaccine stocks of Ad-IiGP, Ad-YF CME or Ad-YF NS3 in phosphate-buffered saline (PBS) to a final volume of 10 μl. Prior to vaccination, mice were sedated with isoflurane and then injected subcutaneously (s.c) in the right hind footpad with 2 × 10^5^, 2 × 10^6^ or 2 × 10^7^ infectious units (IFU) of the adenoviral construct.

### Viral challenge

#### LCMV Armstrong 53b

Mice vaccinated with Ad-liGP were challenged intraperitoneally (i.p) with 2 × 10^5^ pfu of LCMV Armstrong 53b (provided by M.B.A Oldstone, Scripps Research, La Jolla, CA), diluted in PBS, in a total volume of 300 μl. Three days post challenge spleens were removed and stored (−80 °C) for plaque analysis. For evaluation of resistance to lethal infection, mice were challenged i.c. with a dose of 20 pfu in a volume of 30 μl.

#### LCMV Armstrong Clone 13

Mice vaccinated with Ad-liGP mice were challenged intravenously (i.v) with 2 × 10^5^ or 2 × 10^6^ pfu LCMV Armstrong clone 13 (provided by M.B.A Oldstone, Scripps Research, La Jolla, CA) diluted in PBS, in a total volume of 300 μl. Five or ten days post challenge, respectively, spleens were removed and stored (−80 °C) for plaque analysis.

#### Vaccinia-GP

Mice vaccinated with Ad-liGP were challenged i.p. with 2 × 10^6^ pfu Vaccinia-GP (originally obtained from Dr. D.H.L. Bishop (Oxford University, Oxford, U.K.) via Annette Oxenius (ETH, Zürich, Switzerland)) diluted in PBS, in a total volume of 300 μl. Six days post challenge spleens were removed and prepared for intracellular staining.

#### YF-17D virus

Mice vaccinated with Ad-YF CME or Ad-YF NS3 were challenged intracerebrally (i.c) with 10^4^ pfu YF-17D virus (Stamaril, Sanofi Pasteur; reconstituted as recommended by the manufacturer) diluted in PBS, in a total volume of 30 μl. Mice were deeply anaesthetized during this procedure. Seven days post challenge brains were removed, snap frozen and stored for Immuno Focus assay (IFA).

### Bacterial challenge

Mice vaccinated with Ad-liGP were challenged intravenously (i.v) with a lethal dose of Listeria-GP (provided by Drik Schlüter, Institute für Medizinische Mikrobiologie, Otto-von-Guericke Universität Magdeburg, Magdeburg, Germany) diluted in PBS, in a total volume of 300 μl. The preparation of the infectious dose included overnight growth of the bacterial stock at 37 °C in BHI (brain heart infusion) to induce growth phase, and subsequently a 1 h incubation of 1 ml of this culture in 49 ml of fresh BHI medium. After the 1 hour incubation period, OD_600_ measurement allowed the calculation of the bacterial concentration via a predetermined standard curve. The bacterial dose used for challenge was approx. 0.9 × 10^6^ CFU and was controlled by plate culture. Following challenge, mice were monitored for health condition, weight loss and survival until day three.

### Splenocyte preparation

Spleens were removed aseptically and transferred to Hanks Balanced Salt Solution (HBSS). Single-cell suspensions were obtained by pressing the spleens through a fine steel mesh (70 μm), followed by centrifugation and two washes in HBSS, before re-suspension in RPMI 1640 cell culture medium containing 10% FCS supplemented with NaHCO_3_, 2-ME, L-glutamine, and penicillin-streptomycin.

### Flow Cytometry

Approximately 2 × 10^6^ splenocytes were transferred to U-bottom 96 well microtiter plates and incubated for 5 hours (37 °C, 5% CO_2_) with 70 μl RPMI 1640 cell culture medium (containing 1% L-glutamin, 1% penicillin, 1% streptomycin, 1% 2-mercaptoethanol (2-ME) and 10% fetal calf serum (FBS)), supplemented with 50 μl IL-2 (50 IU/ml), 50 μl Monensin (2 μg/ml) and 30 μl (1 μg/ml) of the relevant peptide for stimulation. Control samples did not receive any peptide. Following incubation the cells were centrifuged (2000 rpm, 3 minutes), washed with FACS buffer with monensin (PBS containing 1% BSA, 0.1% NaN3 and 3 μM monensin) and incubated for 20 minutes (4 °C, dark) with 50 μl FACS/Monensin medium containing the relevant surface antibodies (1:100). Cells were then washed twice with PBS/monensin medium (3 μM monensin in PBS) and fixated in 100 μl 2% paraformaldehyde (PFA) & 100 μl PBS/monensin for 15 minutes (4 °C, dark). Afterwards, cells were washed with FACS/monensin medium and incubated for 10 minutes (20 °C, dark) with 200 μl Saponin medium (PBS containing 0,5% Saponin). Next, the cells were incubated for 20 minutes (4 °C, dark) with 50 μl Saponin medium containing the relevant intracellular antibodies (1:100). The cells were subsequently washed twice with Saponin medium and finally resuspended in FACS/Monensin medium and stored at 4 °C until flow cytrometry analysis. Cell samples were analyzed using FACS LSR II cytometer (BD Biosciences) and the data was analyzed using FlowJo software version 7.6.5 (Tree Star).

### Antibodies

The following flourochrome-conjugated monoclonal Abs were used for flow cytometry: for surface staining: α-CD44–FITC, α-CD8-PerCP–Cy5.5, α-CD44–APC/Cy7, and α-CD107α–ALEXA 488; for intracellular cytokine staining (ICS): α-IFN-γ–APC, α-TNF-α–PE/Cy7, and α-IL-2–PE. All antibodies were purchased from Biolegend as anti-mouse antibodies.

### Peptides

The following peptides were used for stimulation: GP_33-41_ (LCMV), GP_276-286_ (LCMV), YF E_4-12_ (Yellow Fever), YF NS3_268-275_ (Yellow Fever)

### *In vivo* cytotoxicity

Single cell suspensions from B6.SJL (CD45.1) splenocytes were prepared as described and resuspended in 4 ml HBSS. Cells were then pulsed with 2.5 μg/ml of either relevant or irrelevant peptide, for 30 min (37 °C, 5% CO_2_). The different cell populations were shaken every 10 min and were washed in PBS and subsequently resuspended in warm (37 °C) PBS to a final concentration of 1 × 10^7^ cells/ml. The cell populations were then mixed with equal volumes of CFSE (Carboxyfluorescein Succinimidyl Ester) or warm PBS and subsequently incubated for 10 min (37 °C, 5% CO_2_) and shaken every 3 min. The labeling was stopped by the addition of 1/10 of the volume of FBS. Afterwards, cells were left for 2 min on ice before they were washed twice in RPMI containing 10% FBS and once in PBS, followed by filtration through a cell strainer (to avoid clumps). The cells were then centrifuged (1200 rpm, 5 min.), resuspended in PBS and mixed in a 1:1 ratio (one cell population loaded with a relevant peptide (GP33) and labeled with CFSE and the other loaded with an irrelevant peptide and unlabeled). 300 μl of the cell mixture were injected i.v into vaccinated B6 (CD45.2) mice or naïve controls. 16 hours later the spleens were harvested and single cell suspensions were again prepared by pressing the spleens through a 70 μm mesh. The cells were then centrifuged, resuspended in 4 ml HBSS and then 200 μl of each cell suspension was transferred to a U-bottom 96 well microtiter plate for surface staining. Cells were centrifuged (1200 rpm, 5 minutes), resuspended in 50 μl FACS medium (PBS, 10% rat serum, 1% BSA, and 0.1% NaN3) containing the relevant surface antibodies (α-CD45.1-APC, diluted 1:100) and left to incubate for 20 min (4 °C, dark). Next, the cells were washed twice with Wash medium (PBS and 0.1% NaN_3_), fixated in 200 μl 1% PFA and stored in 200 μl FACS medium at 4 °C until flow cytrometry analysis. Cell samples were analyzed using Fortessa cytometer (BD Bioscience) and the data was subsequently analyzed using the FlowJo 7.6.5 software (Tree Star).

The *in vivo* cytotoxicity was calculated using the following formula^56^:

Cytotoxicity = 100 - ({(percentage of relevant peptide-pulsed cells in vaccinated mice/percentage of irrelevant peptide-pulsed cells in vaccinated mice)*100}/{percentage of relevant peptide-pulsed cells in naive mice/percentage of irrelevant peptide-pulsed cells in naive mice}).

### Virus titrations

Viral loads in the spleens of mice challenged with LCMV Armstrong 53b or Clone 13 were determined by an immune focus assay (IFA) using MC57G cells. Viral loads in the brains of mice challenged with YF-17D virus were determined by a Vero (ATCC CCL-81) cell based IFA. In both cases organs were first homogenized in PBS to yield 10% organ suspensions, and viral titers were subsequently determined as previously described^38^,^57^.

### *In vivo* CD8+ T-cell depletion

A combination of two monoclonal antibodies (YTS 169 and YTS 156)^58^ was used for *in vivo* depletion of CD8+ T cells from vaccinated mice prior to and during challenge. Mice to be depleted were injected i.p. with 100 μg of each antibody one day prior to challenge plus 1 and 3 days post challenge.

### Statistical evaluation

GraphPad Prism Software (version 6) was used for the statistical analysis. Quantitative results were compared using a nonparametric Mann-Whitney U-test and a p-value of <0.05 was considered evidence of a statistically significant difference.

## Additional Information

**How to cite this article**: Nazerai, L. *et al*. Early life vaccination: Generation of adult-quality memory CD8+ T cells in infant mice using non-replicating adenoviral vectors. *Sci. Rep.* **6**, 38666; doi: 10.1038/srep38666 (2016).

**Publisher's note:** Springer Nature remains neutral with regard to jurisdictional claims in published maps and institutional affiliations.

## Supplementary Material

Supplementary Figures
