# Supplementary material for: Early life vaccination: Generation of adult-quality memory CD8+ T cells in infant mice using non-replicating adenoviral vectors
Source: Sci Rep. 2016 Dec 8;6:38666. doi: 10.1038/srep38666 (PMC5144143; doi:10.1038/srep38666)
Supplement: Supplementary Figures [file srep38666-s1.pdf]

Early life vaccination:
Generation of adult-quality memory CD8+ T cells in infant mice using non-replicating adenoviral vectors

Loulieta Nazerai, Maria R Bassi, Ida E M Uddback, Peter J Holst, Jan P Christensen and Allan R Thomsen*

Department of Immunology and Microbiology,
University of Copenhagen,
Copenhagen, Denmark

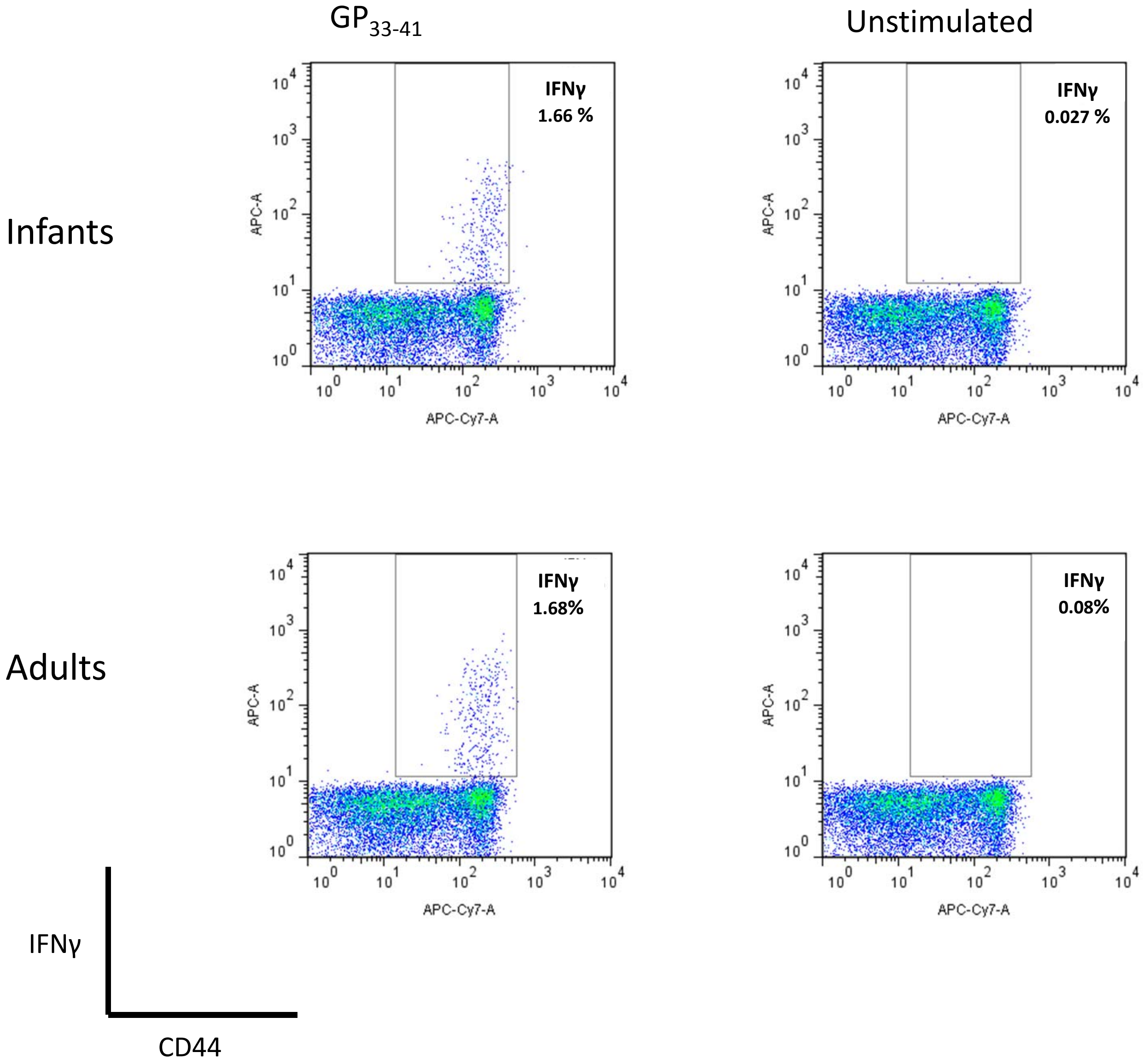

Supplementary figure 1: Evidence for peptide specificity.

Representative dot plots for mice vaccinated with $2x10^7$ pfu Ad-liGP s.c in the foot pad. C57BL/6 mice were vaccinated in the foot pad either as infants or as adults, and 60 days later IFN-γ producing CD8+ T cells were revealed by ex vivo peptide stimulation with GP_{33-41} followed by intracellular cytokine staining. Cells incubated without peptide stimulation served to document epitope specificity. Representative data are from a single experiments included in fig. 1B.

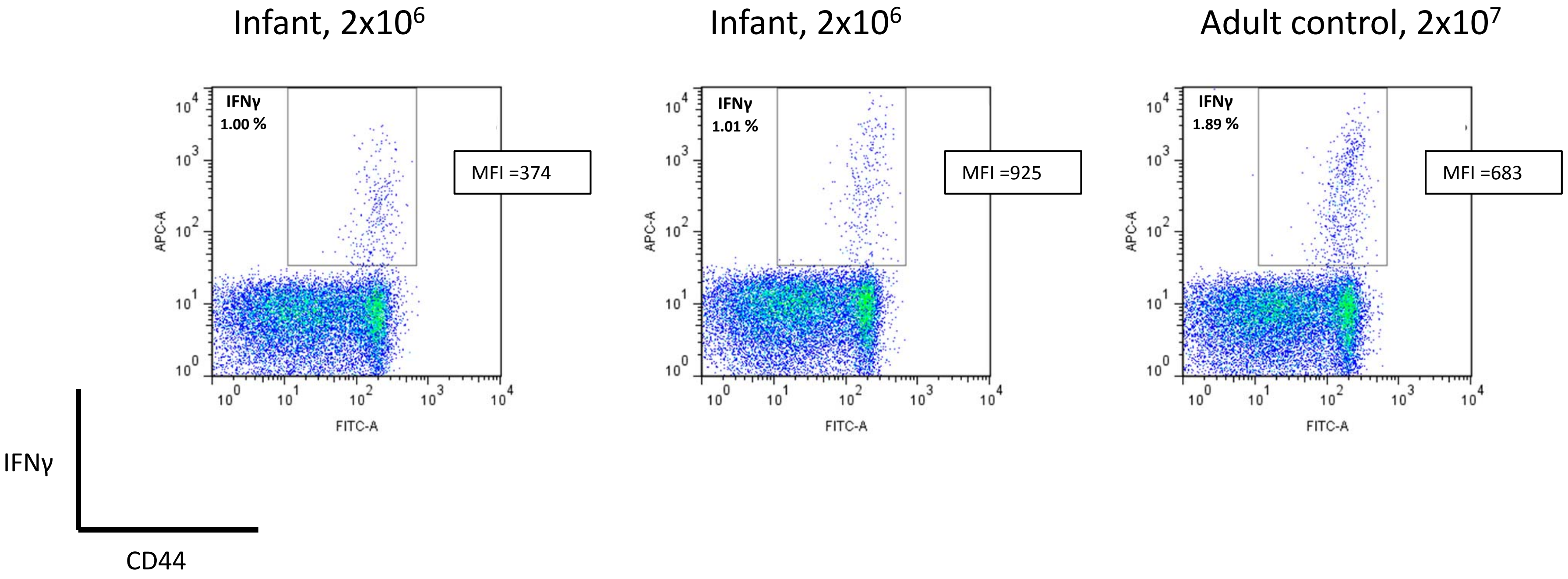

Supplementary figure 2: Evidence for interindividual variation in mean fluorescence intensity (MFI) of cytokine staining of CD8+ T cells from mice vaccinated as infants with $2x10^7$ pfu Ad-liGP s.c in the foot pad.

C57BL/6 mice were vaccinated in the foot pad as described and 60 days later IFN-γ producing CD8+T cells were revealed by ex vivo peptide stimulation with GP_{33-41} and intracellular cytokine staining. Plots representing data from individual mice are depicted. Results from an adult mice vaccinated with $2x10^7$ pfu Ad-liGP s.c in the foot pad have been included for comparison. Representative data are from mice of a single experiment included in fig. 1C.

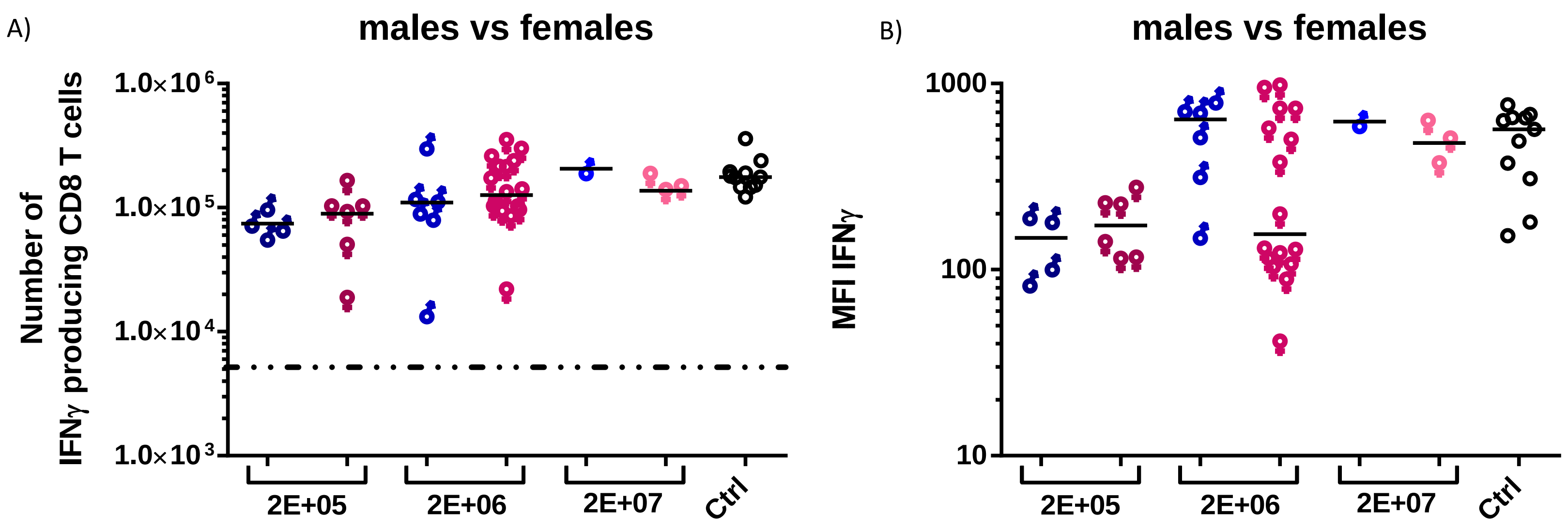

Supplementary figure 3: *No influence of gender on the magnitude and quality of the memory response triggered in Ad-liGP vaccinated infant mice.*

Gender sorting of the results depicted in fig.1. (A) Absolute numbers of CD8+ T cells in the spleen; blue symbols represent males, red symbols females. (B) MFI of the peptide induced IFN-γ signal. Adult female mice, vaccinated with 2x10^7 pfu Ad-liGP, were used as positive controls (Ctrl, black symbols). Each dot represents an individual animal.

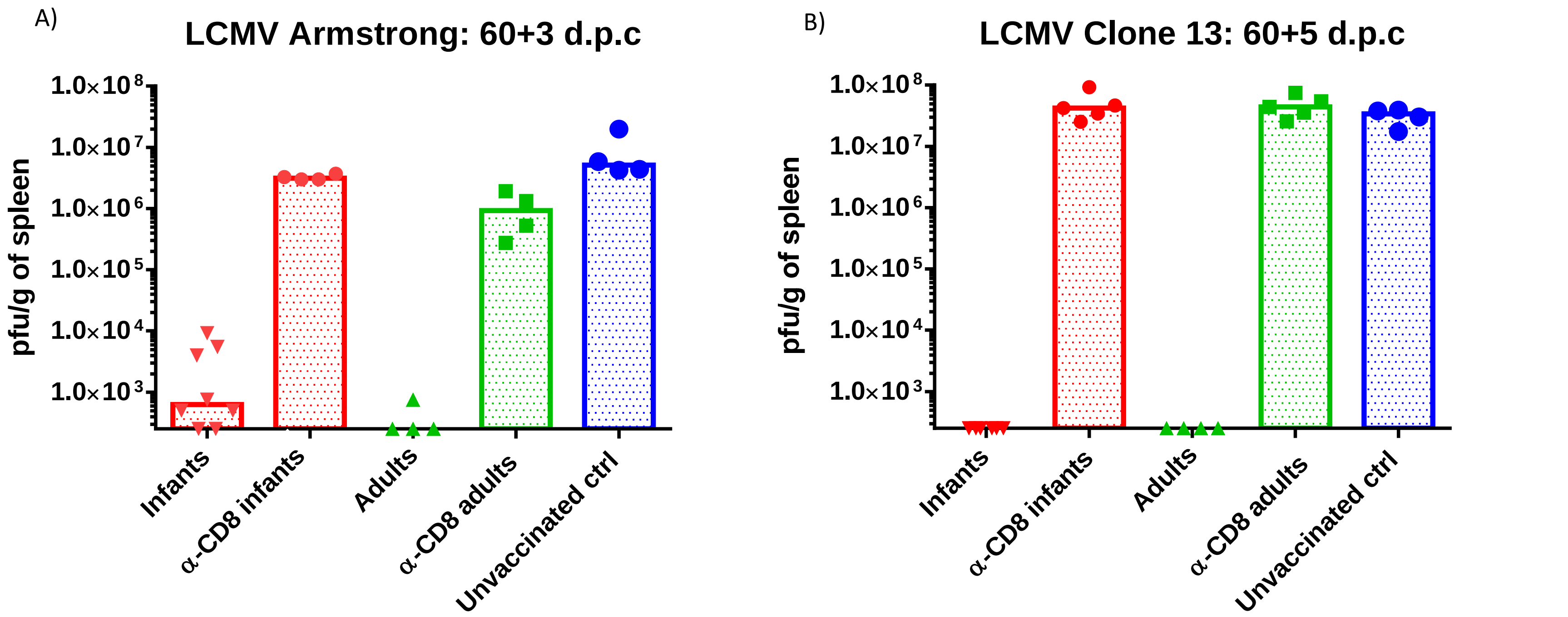

Supplementary figure 4: Vaccine induced antiviral protection is mediated by CD8+ T cells.

C57BL/6 mice were vaccinated with $2x10^7$ pfu Ad-liGP s.c in the foot pad either as infants or as adults. Sixty days later these mice and unvaccinated controls (ctrl) were challenged i.p with $2x10^5$ pfu LCMV Armstrong (A) or $2x10^5$ pfu LCMV Clone 13 (B). Part of the vaccinated mice were depleted of CD8+ T cells immediately before and during challenge, and 3 or 5 days later spleens were removed for analysis of viral content. Each dot represents an individual animal., and bars denote group averages.

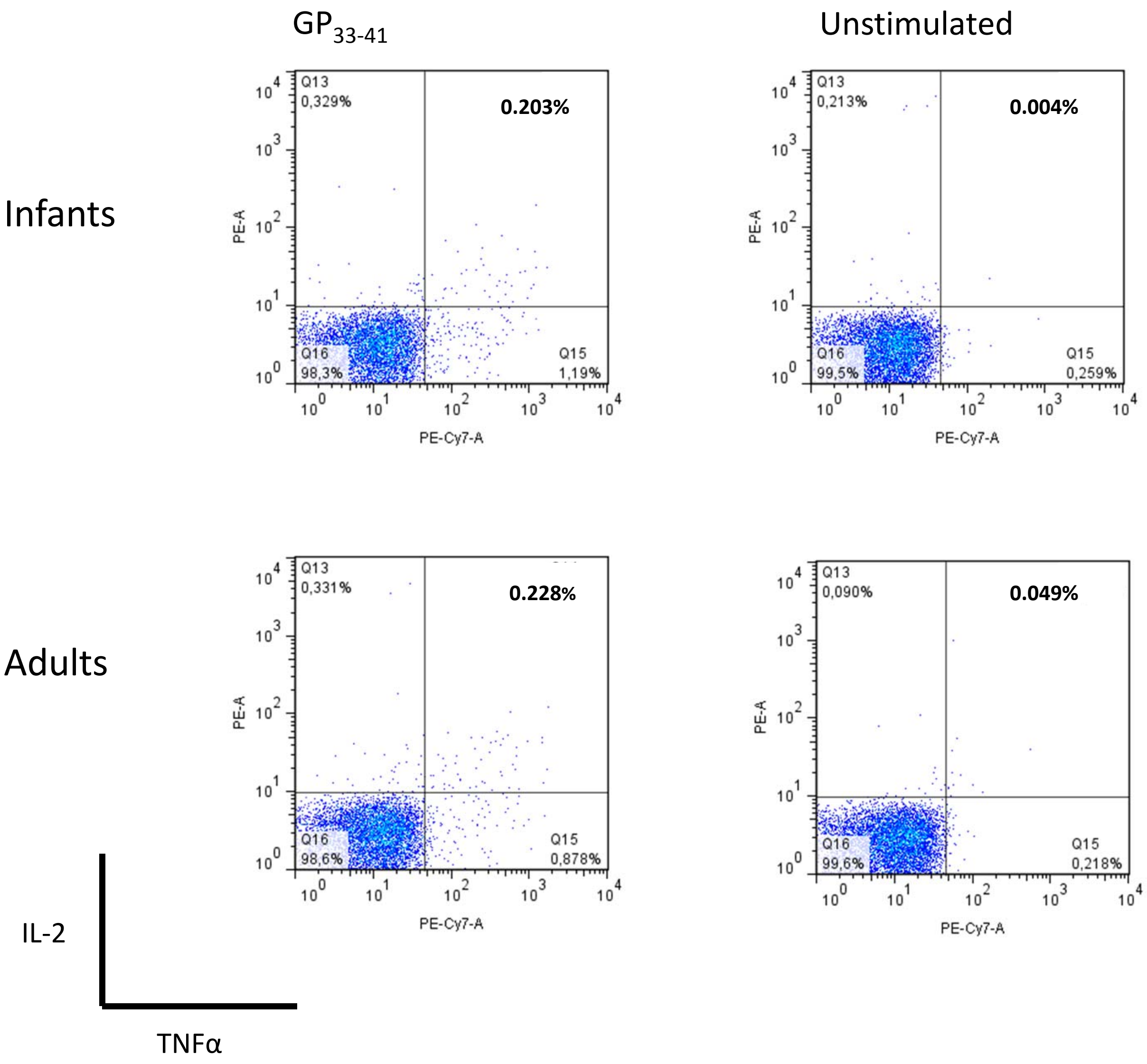

Supplementary figure 5: Capacity of CD8+T cells in Ad-liGP vaccinated mice to co-produce TNF-α and IL-2 is comparable in mice vaccinated as infants or as adults.

C57BL/6 infant mice were vaccinated with $2x10^7$ pfu Ad-liGP s.c in the foot pad and 60 days later the IFN-γ producing CD8+T cells co-expressing TNF-α and IL-2 were measured by flow cytometry following ex vivo peptide stimulation with GP_{33-41}. Mice, vaccinated with $2x10^7$ pfu Ad-liGP as adults, were used as controls. Representative dot plots are depicted. Data are from mice also depicted in fig. 4.

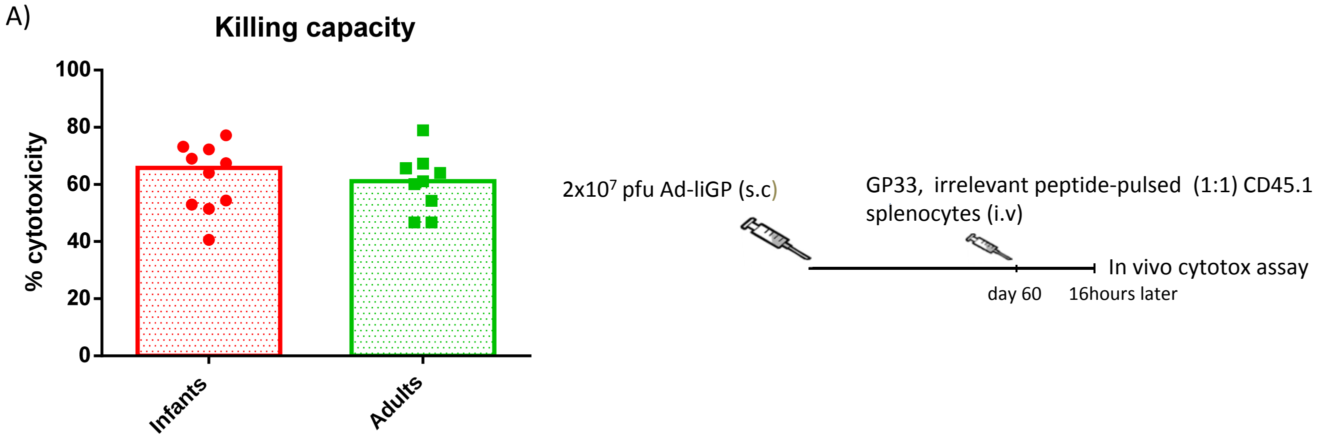

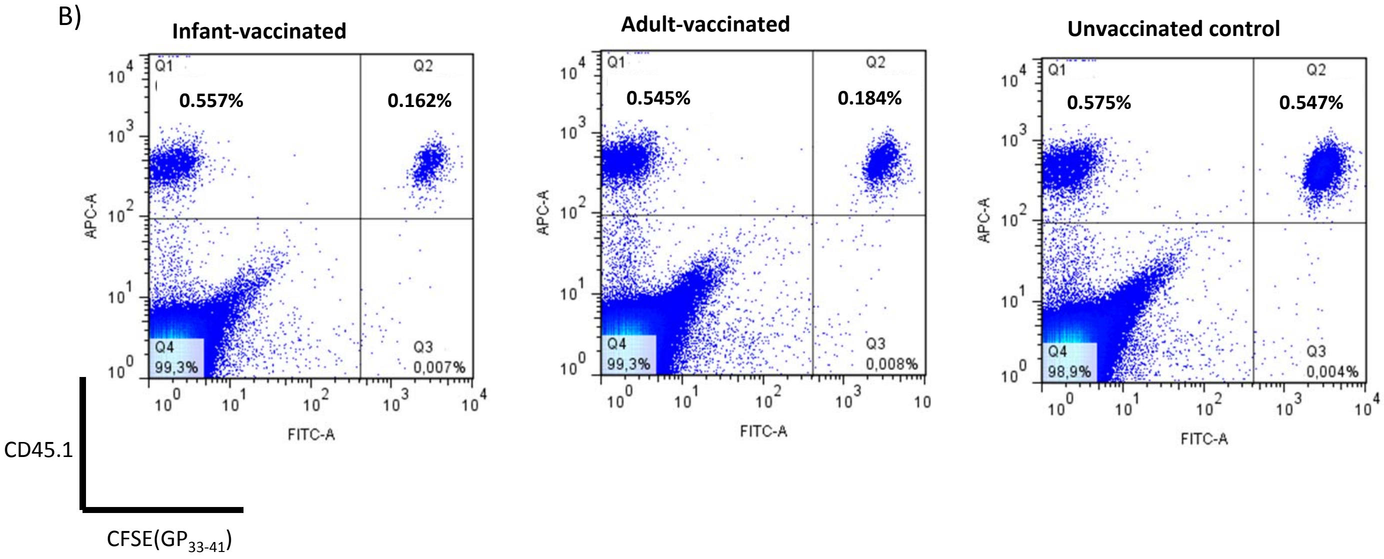

Supplementary figure 6: *The in vivo killing capacity of Ad-liGP primed CD8+T cells is similar regardless of age at vaccination.*

C57BL/6 (CD45.2) infant mice were vaccinated with 2x10^7 pfu Ad-liGP s.c in the foot pad. Sixty days later splenocytes from B6.SJL mice (CD45.1) were pulsed with relevant peptide (GP_{33-41}) or irrelevant peptide. Splenocytes loaded with GP_{33-41} were labeled with CFSE, while the cells loaded with the irrelevant peptide were left unlabeled. The vaccinated mice as well as a group of naïve mice were injected i.v with equal numbers of cells loaded with relevant and irrelevant peptide. After 16 hours, splenocytes from the recipient mice were surface stained with anti-CD45.1 and investigated for CFSE labeling by flow cytometry. Using the formula given in the Materials and Methods section, the in vivo cytotoxicity against GP33 was calculated (A). Mice, vaccinated with 2x10^7 pfu Ad-liGP as adults, were included for comparison. Each dot represents an individual animal. Results are pooled from two independent experiments. Representative dot plots are depicted in (B).

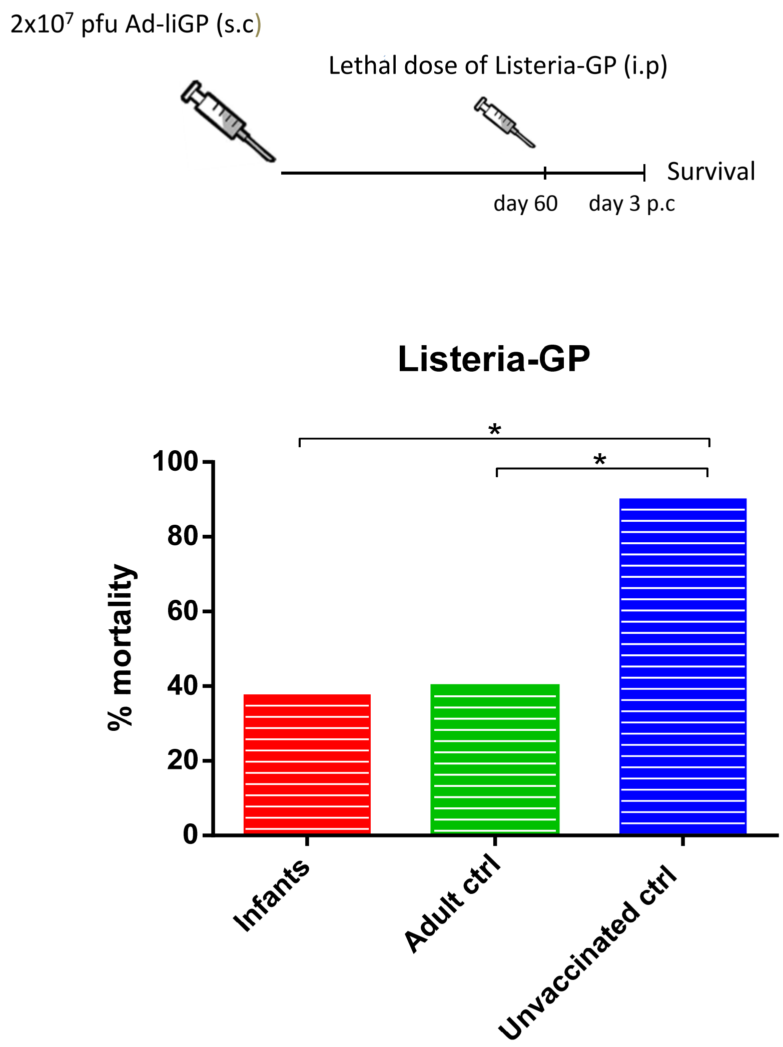

Supplementary figure 7: *Mice are protected from lethal bacterial infection following vaccination with Ad-liGP as infants.*

C57BL/6 infant mice were vaccinated with 2x10^7 pfu Ad-liGP s.c in the foot pad and 60 days later they were challenged with a lethal dose of Lm-GP. The survival of the challenged mice were monitored until day 3. Mice, vaccinated with 2x10^7 pfu Ad-liGP as adults (adult ctrl), and naive unvaccinated mice were included for comparison (ctrl). n= 5-10/group, *p< 0.05
